# Testicular histological and immunohistochemical aspects in a post-pubertal patient with 5 alpha-reductase type 2 deficiency: case report and review of the literature in a perspective of evaluation of potential fertility of these patients

**DOI:** 10.1186/1472-6823-14-43

**Published:** 2014-05-23

**Authors:** Lavinia Vija, Sophie Ferlicot, Diana Paun, Hélène Bry-Gauillard, Gabriela Berdan, Issam Abd-Alsamad, Marc Lombès, Jacques Young

**Affiliations:** 1 Faculté de Médecine Paris-Sud, Univ Paris-Sud, UMR-S693, Le Kremlin-Bicêtre F-94276, France; 2 Inserm, U693, Le Kremlin-Bicêtre F-94276, France; 3 “Carol Davila” University of Medicine and Pharmacy, Bucharest, Romania; 4 Service de Biophysique et Médecine Nucleaire, Assistance Publique-Hôpitaux de Paris, Hôpital de Bicêtre, Le Kremlin-Bicêtre F-94275, France; 5 Univ Paris-Sud, Assistance Publique-Hôpitaux de Paris, Service d’Anatomo-Pathologie, Hôpital Bicêtre, Le Kremlin-Bicêtre F-94276, France; 6 Service d’Endocrinologie et maladies de la Reproduction, Assistance Publique-Hôpitaux de Paris, Hôpital de Bicêtre, 78, rue du Général Leclerc, Le Kremlin-Bicêtre F-94275, France; 7 Department of Pathology,” Burghele” Hospital, Bucharest, Romania; 8 Service d’Anatomo-Pathologie, Centre Hospitalier Intercommunal de Creteil, Creteil F-94276, France

**Keywords:** 5α-reductase type 2 deficiency, Testicular histology, Sertoli cell, Anti-Müllerian hormone, Androgen receptor

## Abstract

**Background:**

Testicular morphology and immunohistochemical studies have never been reported in genetically documented adult patients with 5 alpha-reductase type 2 deficiency (5α-R2 deficiency).

**Case presentation:**

We describe the testicular histopathology of a 17-year-old XY subject with 5α-R2 deficiency caused by the recurrent homozygous Gly115Asp loss of function mutation of the *SRD5A2* gene.We also performed an immunohistochemical analysis in order to further study the relationship between seminiferous tubules structure, Sertoli cell differentiation and androgenic signaling impairment in this case. We thus evaluated the testicular expression of the anti-Müllerian hormone (AMH), androgen receptor (AR) and 3β-hydroxysteroid dehydrogenase (3βHSD). Histological analysis revealed a heterogeneous aspect with a majority (92%) of seminiferous tubules (ST) presenting a mature aspect but containing only Sertoli cells and devoid of germ cells and spermatogenesis. Focal areas of immature ST (8%) were also found. Testicular AR and 3βHSD expression were detected in adult male control, 5α-R2 deficiency and CAIS subjects. However, AMH expression was heterogeneous (detectable only in few AR negative prepubertal ST, but otherwise repressed) in the 5α-R2 deficiency, conversely to normal adult testis in which AMH was uniformly repressed and to an adult CAIS testis in which AMH was uniformly and strongly expressed.

**Conclusion:**

Intratesticular testosterone can repress AMH by itself, independently of its metabolism into dihydrotestosterone. We also compare our results to the few post pubertal cases of 5α-R2 deficiency with available histological testicular description, reported in the literature. We will discuss these histological findings, in the more general context of evaluating the fertility potential of these patients if they were raised as males and were azoospermic.

## Background

The 46, XY disorders of sex development (DSD) are presently classified in three main categories [1]: disorders of gonad development such as gonadal dysgenesis, disorders of androgen biosynthesis and metabolism and disorders related to androgen sensitivity (the androgen insensitivity syndrome, AIS). Within the second category, genetic causes have been identified, such as loss of function mutations of the LH receptor gene (*LHCGR*), 17β hydroxysteroid dehydrogenase deficiency type 3, related to mutations of the *HSD17B3* gene and loss of function mutations of the *SRD5A2* gene, responsible for 5α-reductase type 2deficiencywhich is one of the three 5α-reductase isoforms expressed in humans [2]. In the third category, androgen receptor (*AR*) mutations have been identified in patients with mild, partial or complete androgen insensitivity syndromes (AIS) [1].

AIS have variable phenotypic presentations, mainly related to the severity of the deleterious effects of AR mutations. Indeed, subjects with complete androgen insensitivity (CAIS) present as girls and women with feminine aspect of external genitalia but with absent pubic hair, blind ending vagina and uterine agenesis. In partial androgen insensitivity syndrome (PAIS) phenotypes, there are varying degrees of masculinization, ranging from perineoscrotal hypospadias to minor forms of male infertility, more or less associated with gynecomastia, undervirilization, hypotrophic gonads and micropenis [1].

5α-R2 deficiency phenotype is also variable, ranging from a complete female phenotype at birth to a more or less complete virilization of genitalia [3]. In girls with 5α-R2 deficiency, spontaneous virilization occurring at the onset of puberty usually reveals the condition [3].

In both pubertal and adult subjects with CAIS and 5α-R2 deficiency, very few studies of testicular histology showed severely altered spermatogenesis [4,5]. The severe spermatogenesis impairement in CAIS and 5α-R2 deficiency could be related in part to the bilateral cryptorchidism [6] found in both conditions, but also by either altered levels of testicular testosterone action or the lack of metabolic activation to a more active androgen due to mutated 5α-reductase type 2.

An interesting cellular marker of Sertoli cells, used for the evaluation of the androgen signaling within seminiferous tubules is the anti-Müllerian hormone (AMH). AMH expression is usually recognized as a marker of differentiation of Sertoli cells and considered to be negatively regulated by androgens at puberty and adulthood [7,8].

The first objective of this work was to evaluate the contribution of testicular testosterone versus its 5α-reduced metabolite, dihydrotestosterone (DHT), on AMH repression after puberty and its relation with spermatogenesis. We, therefore, compared the testicular morphology, as well as the AR and AMH expression in a postpubertal case of genetically demonstrated 5α-R2 deficiency with that of a postpubertal case of CAIS. We also compared our findings to published data describing the histological testicular features of post pubertal individuals considered to be 5α-R2 deficient, but in which the diagnosis was not genetically confirmed. Finally, we discuss the testicular histological findings in the more general context of the fertility potential of these patients in adult life, if they were assigned to a male gender.

## Case presentation

The testicular samples of the 5α-R2 deficiency patient were obtained immediately after the bilateral gonadectomy performed when she was 17-years-old. The patient was a post-pubertal XY female with primary amenorrhea, failure of pubertal breast development and virilization, clitoromegaly and bilateral cryptorchidism, with testes located in the inguinal canals. This phenotype was related to a recurrent [3], homozygous, missense deleterious mutation in the exon 2 of the *SRD5A2* gene (c.344G > A; Gly115Asp). The residual 5α-reductase type 2 activity in cells transfected with this mutant was of less than 0.2% [9] The detailed clinical and hormonal characteristics of this patient (testosterone-T: 7.2 ng/mL (normal range in post-bertal males: 3.5-8.5), DHT: 0.16 ng/mL (0.25-1.1), FSH:14.5 IU/L (2.7-7.4), LH: 4.2 IU/L (2.6-6.5), AMH: 65 pmol/L (15–89) as well as the results of the pelvic magnetic resonance imaging (MRI) have been previously described [10].

We comparatively analyzed the testicular histological aspect of this case with those of a CAIS and in a man with obstructive azoospermia but normal testicular spermatogenesis (see below). The CAIS patient was an 18-year-old, XY female, with primary amenorrhea (FSH: 3 IU/L, LH:18 IU/L) and absent axillary and pubic hair and high testosterone (19 ng/mL) and AMH (170 pmol/L) levels. On clinical and ultrasound examinations were observed a blind ending vagina, absent uterus and bilateral cryptorchidism (the left gonad was situated in the pelvis while the right one was located in the inguinal canal). In this patient, the diagnosis of CAIS was confirmed by the identification of a missense mutation in the exon 5 of the *AR* gene. This recurrent, loss of function mutation (c.2194G > A, p.Asp732Asn or D732N upon a previous classification), has already been reported in several CAIS patients [11] and its deleterious character has been previously demonstrated, as functional analysis revealed that this mutant had lost 95% of the wild-type transcriptional activity [11].

The testicular sample, used as a normal control, was obtained from a 29-year-old patient, with normal testosterone and gonadotropin levels (T: 6.8 ng/mL, FSH: 4.5 IU/L, LH: 4.2 IU/L, AMH: 33 pmol/L) undergoing testicular biopsy for obstructive azoospermia. Indeed, for ethical reasons, it was not possible to perform testicular biopsies in healthy men. However, as already reported, more than 86% of patients with obstructive azoospermia present normal spermatogenesis on testicular biopsies [12]. For this patient, the histological analysis of the testicular biopsy showed, as expected, normal structures and ongoing spermatogenesis within seminiferous tubules.

### Histology and immunohistochemistry

All subjects gave their informed written consent to allow the study of testicular samples. This study was approved by the corresponding local ethics committees and was in accordance with the French Bioethics law No.2004-800. All paraffin samples from the three patients were studied by the pathologist (SF), without knowledge of the underlying conditions. For each sample, testicular morphology was assessed on haematoxylin-eosin staining, followed by immunohistochemical analysis for the expression and localization of AR, AMH and 3β-hydroxysteroid dehydrogenase (3βHSD). The immunohistochemistry techniques used were previously reported in detail [13-15]. Immunonegative controls were performed substituting primary antibodies with corresponding preimmune immunoglobulins from the same species. These sections were all immune-negative.

#### Macroscopic aspects

The adult testicular samples used as a control, were harvested from testes with intrascrotal localization, presenting normal volume (18 cm^3^) and morphology. Macroscopically, both testes of the patient with 5α-R2 deficiency exhibited a similar hypotrophic aspect. Thus, the left testis had a 6 cm^3^ volume and weighted 7 g while the right testis volume was 7 cm^3^ and weighted 7 g. The testicular samples of the patient with CAIS obtained after bilateral gonadectomy, exhibited similar size (11 and 9 cm^3^) and macroscopic aspect than the 5α-R2 deficiency patient’s testis.

#### Histological aspects

**Figure 1:**
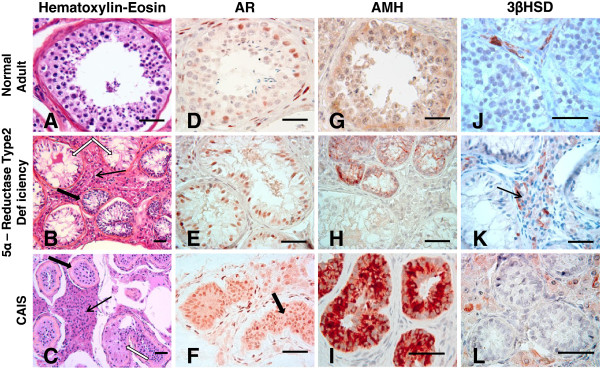
**Histological characteristics and immunohistochemical detection for AR, AMH and 3βHSD expression, in testicular paraffin-embedded sections obtained from a 29 year old human adult with normal testicular structures (Figure **1**: panels A, D, G, J), a 17 year-old teenager with 5 α-R2 deficiency (Figure **1**: panels B, E, H, K) and a 18 year-old CAIS teenager (Figure **1**: panels C, F, I, L).** Panels **A**, **B**, **C**- histology of testicular samples on haematoxylin-eosin staining. Panel **A** shows complete spermatogenesis in a seminiferous tubule section of the normal adult testis; Panel **B** shows heterogeneous seminiferous tubules (ST), in the 5 α-R2 deficiency, such as tubules presenting large diameters and lumina(white arrows) and other tubules with small diameter lacking lumina (thick black arrow); Panel **C**, shows, in a CAIS patient, used for comparison, a homogeneous pattern, with small diameter, immature STs. In this CAIS patient, STs are delineated by a thickened basal membrane (thick black arrow). In one ST, Sertoli cells with an oncocytic transformation of the cytoplasm are indicated (white arrow).The interstitial compartment contains an area of Leydig cell hyperplasia (thin black arrow). Panels **D**, **E**, **F**- show AR immune detection in testicular sections obtained from the three subjects. AR is immunodetected in Sertoli cells, Leydig cells and peritubular myoid cells. Panel **F** evidentiates positive AR immunostaning in ST of the CAIS patient's testicular sample. Panels **G**, **H**, **I**- show AMH immune detection in STs of the testicular sections obtained from the three subjects. Panels **J**, **K**, **L**- show 3βHSD immune detection in Leydig cells of the testicular sections obtained from the three subjects. Scale bars -50 μm.

Haematoxylin-eosin stained paraffin sections from the adult testicular biopsy showed large seminiferous tubules, outlined by spindle-shaped peritubular myoid cells. All seminiferous tubules contained mature Sertoli cells lining the basal membrane and ongoing spermatogenesis (Figure 1A).

**Figure 2:**
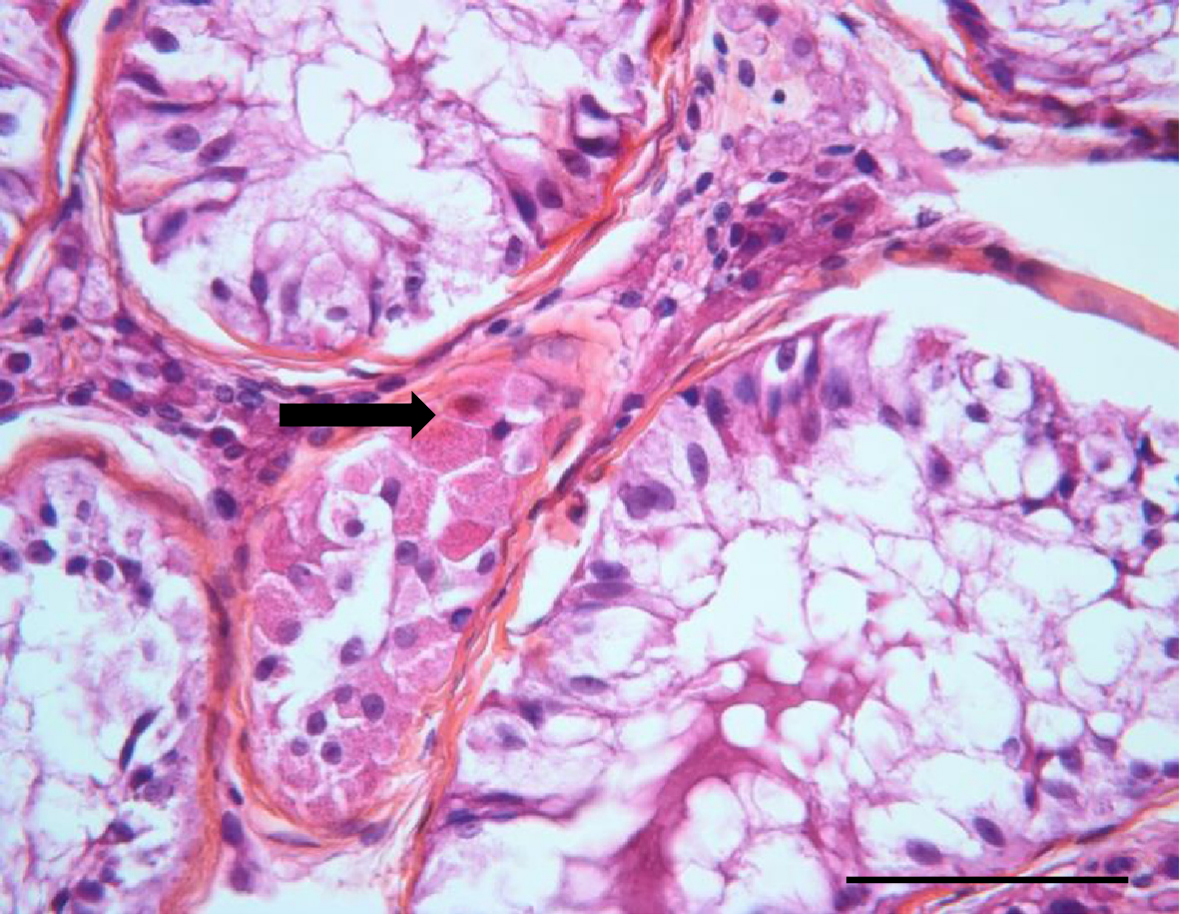
**Testicular section magnification, on hematoxylin-eosin staining, of the 17 year-old teenager with 5 α-R2 deficiency.** Seminiferous tubules with large diameter and containing only Sertoli cells, surround a flattened section of a tubule, containing Sertoli cells with oncocytic transformation of the cytoplasm (thick black arrow). Scale bar-50 μm.

In the 5α-R2 deficiency patient’s testis, haematoxylin-eosin staining revealed heterogeneous seminiferous tubules, surrounded by few peritubular myoid cells and a thickened basement membrane (Figure 1B). The majority of these seminiferous tubules displayed central lumina and normal diameters for the age. They contained two types of Sertoli cells: either mature Sertoli cells, with limited borders and visible nucleoli, or involuting Sertoli cells, with lobulated shapes, irregular borders and inconspicuous nucleoli (Figure 1B). Besides these mature tubules, there was a focal area of seminiferous tubules, representing 8% of the seminiferous tubules, with small tubular diameters and lack of central lumina consistent with an immature, prepubertal pattern (Figure 1B, thick black arrow). These immature seminiferous tubules were characterized by a pseudostratified distribution of Sertoli cells; this aspect was similar to the testicular histology described by Regadera et al*.*[16] in patients with secretory azoospermia caused by hypogonadotropic hypogonadism. Some prepubertal seminiferous tubules also contained few spermatogonia. In order to specify the nature of Sertoli cells histological aspects in this 5α-R2 deficiency patient, we performed a selected magnification (Figure 2). We thus observed some seminiferous tubules, with flattened aspect, where Sertoli cells exhibited an oncocytic cytoplasm (Figure 1B, white arrow and Figure 2, black arrow). The interstitial space in this sample contained large areas of Leydig cells presenting an intense eosinophilic and granular cytoplasm and single round nuclei, consistent with Leydig cell hyperplasia (Figure 1B, thin black arrow).

In the CAIS patient, the testicular sample stained with haematoxylin-eosin showed dispersed seminiferous tubules with a homogeneous immature aspect characterized by small tubular diameters and lack of central lumina. All seminiferous tubules were surrounded by a thickened basal membrane (Figure 1C, thick black arrow) and by peritubular myoid cells. The majority of seminiferous tubules in this testicular sample contained exclusively Sertoli cells with a pseudostratified distribution, consistent with immature seminiferous tubules [16]. Spermatogenesis was absent in all seminiferous tubules in this CAIS subject; spermatogonia were present only in few tubules (data not shown). Likewise the testicular sample of the 5α-R2 deficiency patient, we observed few seminiferous tubules containing several Sertoli cells with an oncocytic aspect (Figure 1C, white arrow). Finally, the interstitial space in the CAIS testis contained focal areas of important Leydig cell hyperplasia (Figure 1C, thin black arrow).

#### Immuno-histochemical studies

To clarify the relationship between abnormal Sertoli cell differentiation, androgen signaling and spermatogenesis defect, we studied the expression of two Sertoli cell markers, AR and AMH in testicular sections in both 5α-R2 deficiency and CAIS patients compared to the control sample (Figure 1, panels D to I).

The AR protein was expressed in Sertoli, Leydig and peritubular myoid cells in the control adult testis sample (Figure 1D) in accordance to previous data reported by us and others [7,8,15]. Similarly, AR was expressed in Sertoli, Leydig and peritubular myoid cells in both 5α-R2 deficiency (92% of tubules) and CAIS (100% of tubules) testes (Figure 1, panels E, F). However, in the 5α-R2 deficiency patient’ testis, the AR immuno-expression was absent in a minority (8%) of seminiferous tubules, with a prepubertal aspect.

As previously reported [7,13,17,18], AMH was barely detected in the normal adult seminiferous tubules with ongoing spermatogenesis (Figure 1G). In the 5α-R2 deficiency patient’s testis, AMH expression was heterogeneous, with no detectable immunostaining in 92% of tubules with larger lumina and AR expression. However, AMH was clearly expressed in focal areas (8% of tubules) corresponding to immature tubules with no AR expression (Figure 1H).

Contrasting with the morphological aspect of the 5α-R2 deficiency patient’s testis, all seminiferous tubules studied in the CAIS patient intensively expressed AMH (Figure 1I). This AMH expression pattern was similar to those previously described in CAIS patients by many groups [7,18].Finally, in order to specify the Leydig cell steroidogenic status, we also evaluated the 3β-hydroxysteroid dehydrogenase (3βHSD) expression by immunostaining (Figure 1, panels J-L). Positive immunolabeling within interstitial Leydig cells was observed not only in the normal adult testicular sample (Figure 1 panel J), but also in the 5α-R2 deficiency case (Figure 1 panel K) as well as in the CAIS sample (Figure 1 panel L).

## Conclusions

Herein, we report the histological and immunohistochemical testicular analysis of a posptubertal patient with 5α-R2 deficiency; the diagnostic was considered in front of the clinical presentation, associated with a high T/DHT ratio, and confirmed by the genetic analysis, which revealed a homozyguous missense mutation in the *SRD5A2* gene (c.344G > A; Gly115Asp). The deleterious character of this recurrent mutation was clearly demonstrated by Wigley et al. [9] who showed a 99% loss of the enzymatic activity of the mutated protein. To our knowledge, we present the first testicular histological description in a postpubertal patient, with a genetic confirmation of the 5α-R2 deficiency. Indeed, the eight previous publications (Table 1) describing with more or less details the testicular histology in postpubertal patients, putatively affected by this pathology, are relatively old and lack any information related to patients *SRD5A2* gene analysis [5,19-25].

**Table 1:** Phenotypical and histological characteristics in postpubertal patients with 5α-reductase type 2 deficiency

**Patient number**	**Age (years)**	**Caryotype**	**Clinical presentation**	TV (ml)/TW (g)*	FSH (IU/L)/(xULN)**	**T (ng/ml)**	T/DHT ratio***	***SRD5A2*** **sequencing**	**Histology**	**Reference (publication year)**
1	14	46,XY	Female PA; cryptorchidism	7/NA	20.0/(2.0)	3.6	36	NA	SGA	[22] (1986)
2	16	46,XY	Female	NA	38.3/(5.0)	NA	NA	NA	PST (1%)	[5] (1999)
				PA; PPH;						SCA (14%)	
			clitoromegaly								
			cryptorchidism^§^						SGA (25%)		
									SCO (60%)		
3	16	46,XY	Female	3/ 15.5	1.0/(0.5)	5.8	29	NA	SCA	[22] (1986)	
			PA; PPH; 4 cm phallus						SGA		
			cryptorchidism^§^						SCO		
4	16	46,XY	F emale PA; PPH; 3 cm phallus cryptorchidism	11/ 22	4.5/NA	11.4	34.5	NA	Normal	[22] (1986)	
5	16	46,XY	Female PA; cryptorchidism	NA	8.7/(1.0)	9.0	225	NA	SGA	[22] (1986)	
6	17	46,XY	Female PA; PPH; 3 cm phallus	3/NA	20/(1.0)	7.2	NA	NA	SCO	[24] (1980)	
7	18	46,XY	Female PA; PPH; 3 cm phallus	3/NA	56/(3.1)	6.8	NA	NA	SCO	[24] (1980)	
8	18	46,XY	Female PA; PPH; 1.5 cm phallus	15/NA	5.5/(1.1)	10	39	NA	Normal	[19] (1980)	
9	18	46,XY	Female PA; PPH; clitoromegaly cryptorchidism^§^	NA	9.0/(1.3)	NA	NA	NA	PST (10%)	[5] (1999)	
									SCO (90%)		
10	18	46,XY	Female PA; PPH; clitoromegaly	NA	13.0/(2.0)	NA	NA	NA	PST (4%)	[5] (1999)	
									SCO (96%)		
11	25	46,XY	Female PA; PPH; 3 cm phallus	NA	NA	11	42	NA	Normal	[23] (1979)	
12	35	46,XY	Female PA	8/NA	32.0/(3.2)	6.7	33.5	NA	Normal SCO	[22] (1986)	
13	45	46,XY	NA	NA	NA	NA	NA	NA	Normal	[25] (1977)	
14	65	46,XY	Male perineal hypospadias; 6 cm phallus cryptorchidism	NA	40/(4.0)	5.9	38	NA	SCO (100%)	[21] (1980)	
15	NA	46,XY	NA	NA	NA	NA	NA	NA	Normal	[20] (1982)	
**Our case**	17	46,XY	Female PA; PPH; clitoromegaly cryptorchidism	9/ 8	14.5/(2.0)	7.2	45	Gly115Asp	PST (8%)	present paper	
									SCO (92%)		

^*^TV /TW: mean testicular volume in mililiters/ mean testicular weight in grams; ^**^(xULN): ratio between the measured value and the upper limit of normal for the corresponding FSH assay; ***T/DHT ratio: testosterone/dihydrotestosterone ratio; the ratio cutoff for the diagnosis of 5α-reductase type 2 deficiency was set at 10 [3].

PA: primary amenorrhoea; PPH: poseudovaginal perineoscrotal hypospadias; ^§^For these individuals cryptochidism was not specified in the references, but it was suspected, given the female phenotype; NA: not available.

SGA: spermatogenic arrest at the level of spermatogonia; PST: prepubertal seminiferous tubules; SCA: spermatogenic arrest at the level of spermatocytes; SCO: Sertoli cell only; Normal: normal spermatogenesis.

Overall, histological features in those postpubertal patients with 5α-R2 deficiency seem to be rather heterogeneous, varying from complete lack of spermatogenesis to apparently normal spermatogenesis (Table 1).

The histological analysis of the testicular samples obtained from our patient revealed a heterogeneous aspect with a wide majority of seminiferous tubules presenting a mature aspect but a severely altered spermatogenesis, and containing only Sertoli cells. In a minority of seminiferous tubules of prepubertal appearance, where Sertoli cells exhibited a pseudostratified distribution, few germ cells were identified. Overall, the histological aspect of our patient’s testicular samples was similar to that described by Steger et al. [5], in three postpubertal patients (Table 1, patients 2, 9 and 10), presenting with clinical features resembling a 5α-R2 deficiency. The combined histological study of our case, and the analysis of the available literature (Table 1) also showed a strong percentage of impaired spermatogenesis (7/8, 87%) in cryptorchid 5 alpha-reductase type 2 deficiency patients [5,21,22]. These findings suggest that cryptorchidism by itself could be deleterious for spermatogenesis. Similarly, we compared serum dihydrotestosterone (DHT) levels in subjects with histologically normal spermatogenesis with those measured in patients presenting with impaired spermatogenesis (Table 1). Within the first group of patients (n = 4), mean DHT levels were of 0.26 ± 0.05 ng/mL whereas in the second group mean DHT levels were 0.12 ± 0.07 ng/mL. We therefore noticed a trend toward reduction in serum DHT levels in patients with impaired spermatogenesis suggesting that a more severe impairment in 5 alpha-reductase type 2 activity could also contribute to the spermatogenesis alteration.

Histological analysis of the testicular samples of the 5α-R2 deficiency patient reported here also revealed an oncocytic transformation of Sertoli cell cytoplasm in very few seminiferous tubules. This minoritary peculiar histological feature is similar to that described in several infertile subjects [26].The pathophysiological significance of this feature is still unknown, but it remains to be determined whether this oncocytic transformation may be associated to testis tumor development. Important areas of Leydig cell hyperplasia were also observed in our case; such morphological observation has been previously found in 5α-R2 deficient testis [22,24,25].

Similarly, Leydig cell hyperplasia has been already reported in CAIS postpubertal patients [4], or in men with Klinefelter syndrome [27]. In all these different pathological conditions, Leydig cell hyperplasia could be related to chronic Leydig cell stimulation induced by the long-lasting LH excessive secretion [14,24,27,28]. The risk of progression of these lesions to a Leydig cell tumor is unknown in 5α-R2 deficiency but deserves to be monitored if the testes are preserved.

The immunohistochemical analysis also revealed that in our patient, the androgen receptor (AR) was expressed in all three testicular compartments (Sertoli cells, Leydig cells and peritubular myoid cells), suggesting the possibility of a global cellular response to the intratesticular testosterone in this case of 5α-R2 deficiency. Most notably, AR was expressed in 92% of the seminiferous tubules, namely in those with a mature aspect. However, AR expression was below detectable levels in 8% of the seminiferous tubules, specifically those with an immature aspect. Along this line, it is worth noting that AMH immunodetection was rather heterogeneous, as previously reported in a case of 5α-R2 deficiency with “Sertoli cell only” histology [5]. We showed that AMH was repressed in the majority of the seminiferous tubules, whereas AMH was only detectable in immature tubules lacking AR expression. These results suggest that intratesticular testosterone seems sufficient to repress AMH, when AR is expressed, in spite of the 5α-R2 deficiency, and thus irrespective of the conversion of testosterone into dihydrotestosterone. The absence of AMH repression in few seminiferous tubules, in which AR was lacking, is in accordance with previous data published [7,8], supporting the need of AR expression in Sertoli cells for AMH repression. Interestingly, in our patient, AMH was repressed in Sertoli cells harboring mature morphology, even in the absence of germ cells. This suggests that testosterone could exert direct inhibitory effects on AMH expression, independently from spermatogenesis initiation [29]. Alternatively, the absence of spermatogonia in most seminiferous tubules of our patient might have led to functional alterations in Sertoli cells, disrupting their ability to synthesize AMH.

In our patient with 5 alpha-Reductase type 2 Deficiency, serum AMH level was within normal range for postpubertal male as reported in few cases with this condition [30]. This contrasts with the very high serum AMH levels we observed in our CAIS patient, which were in line with the increase in AMH levels reported in adult CAIS patients [31]. This difference in serum AMH levels in 5 alpha-Reductase type 2 Deficiency versus CAIS reinforces the hypothesis that testosterone conversion into dihydrotestosterone seems not essential for AMH repression.

Identification of various spermatogenesis stages in patients with 5α-R2 deficiency is of importance in the context of sex assignment and fertility preservation. Indeed, due to recent progress in the assisted reproduction techniques, successful results with testicular sperm extraction (TESE) and intracytoplasmic sperm injection (ICSI) are now reported in azoospermic patients, either with androgen insensitivity [14] or with Klinefelter syndrome [27]. Concerning 5α-R2 deficient patients, normal spermatogenesis with spontaneous fertility is extremely rare (two published cases) [32]. On the same way, spermatozoa detection in the seminal fluid is also a very rare event, found in only nine 5α-R2 deficient cases [33-35], allowing either intrauterine insemination of the partner in one case [33], or an in vitro fertilization by ICSI, as recently reported for two patients [34,35]. Except these uncommon cases, fertility remains an important issue for 5α-R2 deficient patients, notably those harboring a severe enzymatic defect that precludes spontaneous parenthood and sperm recovery in the ejaculate (i.e. azoospermic patients). In these latter cases, of unknown prevalence, the only fertility therapeutic possibility could be the testicular sperm extraction (TESE). This therapeutic option seems reasonable since as indicated above, TESE has been used as a successful therapeutic strategy in a number of patients with non obstructive azoospermia of various origins, including those with cryptorchidism [36]. In this context, a detailed testicular histological analysis is crucial since this is currently the only reliable method to know if there is spermatozoa in the testis and if spermatozoon are present, to carry out a cryopreservation for ICSI. Finally, a direct relationship between the *SRD5A2* gene mutation severity and the presence of spermatozoa still remains an unsolved question, opening further studies with regards to the prognostic and risk stratification in relation with the severity of the enzymatic defect.

## Consent

Written informed consent was obtained from the patient with 5α-R2 deficiency, as well as from both the CAIS patient and the individual with obstructive azoospermia, for genetic and histological analyses and for publication of this case report and the accompanying images. Copies of written consents are available upon request for review by the Journal Editor.

## Abbreviations

5α-R2 deficiency: 5α-reductase type 2 deficiency; AIS: Androgen insensitivity syndromes; AMH: Anti-Müllerian hormone; AR: Androgen receptor; 3βHSD: 3β-hydroxysteroid dehydrogenase; TESE: Testicular sperm extraction; ICSI: Intracytoplasmic sperm injection.

## Competing interest

The authors declare that they have no competing interests.

## Authors’ contributions

LV, ML and JY led the conception and design, acquisition of data, review of literature, and drafted the manuscript. HBG and DP critically reviewed the manuscript. SF, IAA and GB provided the paraffin embedded samples and advised on the pathology pictures. All authors read and approved the manuscript.

## Author’s information

LV is PhD student at INSERM-Paris Sud Research Unit U693 and assistant at Department of Biophysics and Nuclear Medecine at Bicêtre Hospital, France. ML is head of the INSERM-Paris Sud Research Unit U693, France. JY is Professor of Endocrinology at Université Paris-Sud and Medical practitioner at the Department of Reproductive Endocrinology, Bicêtre Hospital, France.

## Pre-publication history

The pre-publication history for this paper can be accessed here:

http://www.biomedcentral.com/1472-6823/14/43/prepub
